# Influence of Visual Motion, Suggestion, and Illusory Motion on Self-Motion Perception in the Horizontal Plane

**DOI:** 10.1371/journal.pone.0142109

**Published:** 2015-11-04

**Authors:** Steven David Rosenblatt, Benjamin Thomas Crane

**Affiliations:** 1 Department of Otolaryngology, University of Rochester, Rochester, New York, United States of America; 2 Department of Neurobiology and Anatomy, University of Rochester, Rochester, New York, United States of America; 3 Department of Bioengineering, University of Rochester, Rochester, New York, United States of America; Durham University, UNITED KINGDOM

## Abstract

A moving visual field can induce the feeling of self-motion or vection. Illusory motion from static repeated asymmetric patterns creates a compelling visual motion stimulus, but it is unclear if such illusory motion can induce a feeling of self-motion or alter self-motion perception. In these experiments, human subjects reported the perceived direction of self-motion for sway translation and yaw rotation at the end of a period of viewing set visual stimuli coordinated with varying inertial stimuli. This tested the hypothesis that illusory visual motion would influence self-motion perception in the horizontal plane. Trials were arranged into 5 blocks based on stimulus type: moving star field with yaw rotation, moving star field with sway translation, illusory motion with yaw, illusory motion with sway, and static arrows with sway. Static arrows were used to evaluate the effect of cognitive suggestion on self-motion perception. Each trial had a control condition; the illusory motion controls were altered versions of the experimental image, which removed the illusory motion effect. For the moving visual stimulus, controls were carried out in a dark room. With the arrow visual stimulus, controls were a gray screen. In blocks containing a visual stimulus there was an 8s viewing interval with the inertial stimulus occurring over the final 1s. This allowed measurement of the visual illusion perception using objective methods. When no visual stimulus was present, only the 1s motion stimulus was presented. Eight women and five men (mean age 37) participated. To assess for a shift in self-motion perception, the effect of each visual stimulus on the self-motion stimulus (cm/s) at which subjects were equally likely to report motion in either direction was measured. Significant effects were seen for moving star fields for both translation (p = 0.001) and rotation (p<0.001), and arrows (p = 0.02). For the visual motion stimuli, inertial motion perception was shifted in the direction consistent with the visual stimulus. Arrows had a small effect on self-motion perception driven by a minority of subjects. There was no significant effect of illusory motion on self-motion perception for either translation or rotation (p>0.1 for both). Thus, although a true moving visual field can induce self-motion, results of this study show that illusory motion does not.

## Introduction

Visual field motion can ambiguously be perceived as either self-motion in a fixed environment or as external motion relative to a fixed observer. The visually induced sensation of self-motion is known as vection [1–5].

The perception of visual motion can also be found in the absence of any actual movement. Static repeated asymmetric patterns (RAPs), such as the rotating snakes image [6–8] and the peripheral drift illusion [9], can create the sensation of compelling illusory motion. These images, colloquially referred to as optical illusions, create the perception of motion though the images themselves are static. Our understanding of how these images induce the sense of motion has evolved over the years and an in-depth discussion of this is beyond the scope of this study. In brief, there is some consensus that the illusory motion effect results from timing differences between neuronal responses to different contrast and/or luminance elements, and that static RAPs evoke a similar pattern of neural activity that occurs when objects are actually moving [10, 11]. Previous studies have demonstrated that vection can be induced not only by moving visual stimuli [1, 12–15], but also in the absence of explicit motion with only illusory visual motion [16]. In the Seno et al. study, the illusory motion stimulus produced a greater vection perception than a control stimulus, but the control stimulus still produced some vection, which raises the issue of whether the reported vection was actually equivalent to self-motion. Like many studies of vection[17–21], the Seno et al. study used a magnitude estimation technique,[22] which involved having observers give a subjective numeric value to their perception. However, such subjective reporting makes it difficult to determine if differences in subject responses are due to the underlying perception or to the interpretation of the stimulus in relation to the reporting scale [23–25]. Although this can be partially addressed by reporting average responses for the study population[16], this does not allow potential differences in perception between subjects to be studied. Thus, the nature of this technique does not allow the amount of self-motion associated with the reported vection to be known. Furthermore, because magnitude estimation techniques are often not standardized between studies, it makes it difficult to make comparisons.

Another method of quantifying vection that avoids this issue is to compare vection stimuli to inertial stimuli (actual movement) [1, 26–29]. Although this method has shown that visual motion can bias inertial motion perception, it has not previously been applied to illusory visual motion. Thus, it is unknown if illusory visual motion can alter the perception of inertial self-motion in the same way as the effect of a moving visual field. This study tests the hypothesis that illusory visual motion can be perceived as self-motion in the horizontal plane by looking at effects of these visual stimuli on perception of an inertial stimulus.

## Materials and Methods

### Ethics Statement

Informed written consent was obtained from all participants prior to their participation. The protocol, including the written consent form, used was approved by the University of Rochester Research Science Review Board and conducted according to the principles expressed in the Declaration of Helsinki.

### Visual Stimulus

Visual stimuli were displayed on a color LCD screen measuring 115.6 x 64.8 cm with a resolution of 1920 x 1080 pixels (Samsung model LN52B75OU1FXZA), which was placed 50cm from the viewer and encompassed a 98° horizontal field of view.

### Illusory visual motion

**Fig 1:**
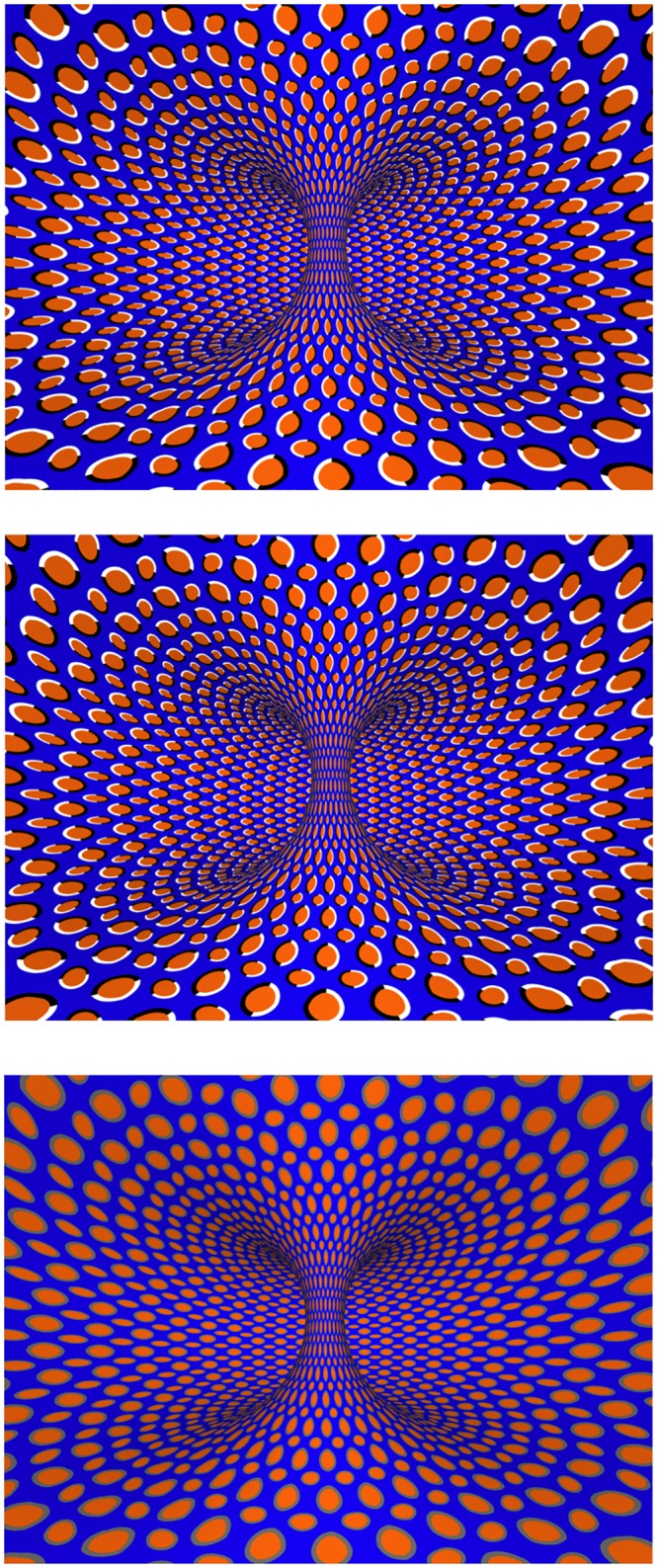
Images used to produce illusory motion. The top image gives the illusion of the inside of torus rotating to the left relative to a fixed observer. The middle image is the top image turned upside down and gives the illusion of rotation in the opposite direction. The bottom image is a control in which the black/white pairs are replaced with gray. The bottom image continues to convey the shape of the inside of the torus without giving the illusion of motion. These images were provided by Beau Dealey.

For the illusory motion visual stimulus, we used a static repeated asymmetric pattern mapped onto a torus (Fig 1, top) that was rendered by Beau Dealy. A mirror image of this stimulus was used for lateral motion in the opposite direction (Fig 1, middle), and a control image with gray circles replacing the black/white pairs was created to eliminate the illusory motion effect but maintain the three-dimensional shape (Fig 1, bottom).

### Actual visual motion

The true motion visual stimulus consisted of a moving star field that simulated movement of the observer through a random-dot cloud, either translating from one side to the other in the leftward or rightward directions, or rotating about the subject. Each star consisted of a triangle 0.5 cm in height and width at the plane of the screen, adjusted for distance. The star density was 0.01 per cubic cm. The stimulus was presented at a constant velocity of 45 cm/s or 45°/s. The depth of the field was 130cm.Visual coherence was fixed at 100%. Disparity was provided using red-green anaglyph glasses made with Kodak (Rochester, NY) Wratten filters #29 (dark red) and #61 (deep green). The colors were adjusted so that the intensities of the two were similar when viewed through the respective filters and the rejection ratio was greater than ten fold.

### Cognitive suggestion

To evaluate whether higher cognitive suggestion played a role in influencing heading perception, we created two static arrow images, one pointing to the right and the other to the left. The arrow was black on a gray background and appeared at eye level. It was 29 cm long and 9 cm high. For a control, a blank gray screen (the same color as the background for the arrows) was displayed.

### Inertial Stimulus

Motion stimuli were given using a 6-degree-of-freedom motion platform (Moog, East Aurora, NY, model 6DOF2000E), which was previously described [30–34]. A padded racing seat (Corbeau, Sandy UT, model FX-1) was mounted to the platform with a four-point racing style harness to hold the subject in place. An American football helmet (Riddell, Eyria, OH) was used to secure the head in place and the facemask was removed to improve visibility. Helmets were available in a variety of sizes and contained an adjustable air bladder to assure a snug fit. A rigid coupling device was attached to the right lateral aspect of the helmet, which was secured to the chair to prevent decoupling or unwanted head motion. The head was positioned such that the body was midline and the center of the platform was between the external auditory canals. An audible white nose stimulus was given during the inertial stimuli to mask the sound of the platform motion, as previously described [30].

The inertial stimulus consisted of a 1s (0.5Hz) sine wave acceleration and occurred in a single direction. Subjects were automatically returned to a center point after reporting the perceived direction of each stimulus. Only left/right sway translation and left/right yaw rotation were tested in this experiment. These motion profiles have been previously used in the current laboratory [30, 31, 33, 35] and by others [36, 37] as they are well accepted in studies such as this. In addition, these motion profiles contain no discontinuities in acceleration, velocity, or position. A small amount of fore-aft mechanical oscillation was added to each test stimulus to minimize non-vestibular cues (e.g. sound and vibration), which may give the subject additional information as to the direction of travel. For the translational experiments, the maximum displacement was 10cm and for the rotation experiment the maximum displacement was 10° of rotation.

### Experimental Procedure

Experiments were arranged into five blocks based on the type of visual stimulus being presented (actual visual motion with a moving star field, illusory motion, and arrows). The blocks included: moving star field with yaw (left/right rotation), moving star field with sway (left/right translation), illusory motion with yaw, illusory motion with sway, and arrows with sway. Because a decision was made to test rotation later, sway experiments were carried out first. The rotation experiments were mostly conducted 1–2 months after the others, except for subject #13 who was enrolled after the rotation experiments were implemented and all of the conditions were completed within a few days. Beyond that, the order of experiments was randomized for each subject. Within each condition, the visual stimuli with their respective leftward and rightward orientations remained unchanged, while the inertial movement was varied based on each subject’s responses.

Once the subject was properly seated and the apparatus engaged, a 500Hz 0.125s tone was played to signal the start. When the center button was pressed by the subject, the visual stimulus (if applicable) was immediately presented. Each experimental condition had a viewing interval of 8s for the visual stimulus. The inertial stimulus was presented during the last second of the viewing interval. After the 1s inertial stimulus, the visual stimulus disappeared and two 500Hz 0.125s tones were played in rapid succession to indicate the stimulus was complete and the perceived direction should be entered. Once a response was given, the chair automatically reset to the start position over 2.5s. If no response was given after 2s, a low frequency “time out” sound was given and no response was logged. The chair then was returned to the start position. No response was entered rarely and occurred in <1% of stimulus presentations. Subjects were encouraged to guess the direction of travel if they were unsure, as this was a forced-choice paradigm. This algorithm has been previously used in the current laboratory [32, 33, 38] and by others [39, 40].

Each block had a unique control condition, and subjects were randomized to start with either the experimental condition or control condition. In the illusory motion and arrow conditions, the control condition contained a unique visual stimulus (Fig 1 bottom or plain gray background with no arrow for the arrow stimuli), which was presented for the same duration as the illusory and visual motion stimuli with the inertial stimulus also coming in the last second of viewing. The control condition for the optic flow stimuli did not have any visual stimulus for the control condition, and the control experiment was carried out in a dark room. Because of this, there was no looking interval prior to the motion stimulus after the center start button was pressed.

Responses were collected using a three-button response box. The subject was instructed on how to hold and use the box prior the experiments. The center button was designated the start button. The left and right buttons corresponded to their respective directions. Once the experiment was initiated, an audible tone was used to indicate the next stimulus was ready, after which the subject would press the center button.

[41]Control conditions consisted of a single staircase of 25 movements, while experimental conditions contained two interleaved independent staircases for left/right visual stimuli of 25 steps each for a total of 50 movements. This was done to minimize subjects’ ability to identify patterns in the stimulus presentation. The algorithm would not allow more than five stimuli in a row in a single direction. Only the inertial stimuli were varied with the staircase. The initial inertial stimuli were at the maximum displacement for each condition (10 cm or 10°) ensuring they were above the subjects’ threshold. Responses were logged using the response box. After each response the subsequent stimulus was shifted towards the direction opposite the response. For instance, after a 10 cm rightward stimulus which was identified as right, the subsequent stimulus was shifted 4 cm to the left such at a 6 cm rightward stimulus was delivered. Each staircase could pass through zero so that stimuli could eventually be presented in the opposite direction. The initial step size was 4 cm or deg. Within a staircase, the step size was decreased by half when the direction of responses reversed to a minimum of 0.25 cm or deg. Likewise, if the response was in the same direction 3 times in a row the step size doubled to the maximum (4 cm or deg). This method has the effect of concentrating responses near the point of subjective equality (PSE) after a relatively small number of responses in order to efficiently measure the mean of the psychometric function.

Subjects were instructed that each visual stimulus would move or simulate motion in a particular plane and they would receive a physical (inertial) motion stimulus in a congruent plane. The subjects understood that the inertial movement only would occur during the final 1s of the stimulus when masking noise[41] was present. They were further instructed that after the stimulus, they should report their perceived direction of self-motion during the masking noise as either left or right using the buttons on their response box. The viewing monitor dominated the subjects’ visual field. The subjects were given a brief practice run to ensure they properly understood the instructions and operation of the experimental mechanism. Once oriented, the subjects completed their assigned experiment block. If more than one block was completed in a session, subjects were given adequate time to rest out of the apparatus between sessions. Typically no more than two blocks were completed in one day. For the majority of subjects, all experiments were completed over the course of a few weeks.

Before starting a block with illusory motion, subjects were instructed to allow their eyes to wander around the image, as fixation is known to negate the illusory motion effect [10]. At 50% completion, the subject was given a second verbal reminder to allow their eyes to wander and not fixate on a point. When using the illusory motion stimuli, subjects were asked if they perceived the illusion.

### Subjects

A total of 13 subjects participated in the experiment. There were eight women and five men. Ages ranged from 20–66 years (mean ± standard deviation 37 ± 19). Subject ages covered a range of the adult population and prior studies have not demonstrated that thresholds of rotation perception vary significantly within this range [30]. All subjects were healthy individuals without any history of vestibulopathy, cognitive deficit, or hearing loss. No subjects were taking vestibular suppressants. Thirteen subjects completed the blocks with sway inertial stimuli. Three subjects were no longer available to participate in the rotation stimuli. The remaining ten went on to complete all blocks including the rotation inertial stimuli.

### Statistical Analysis

**Fig 2:**
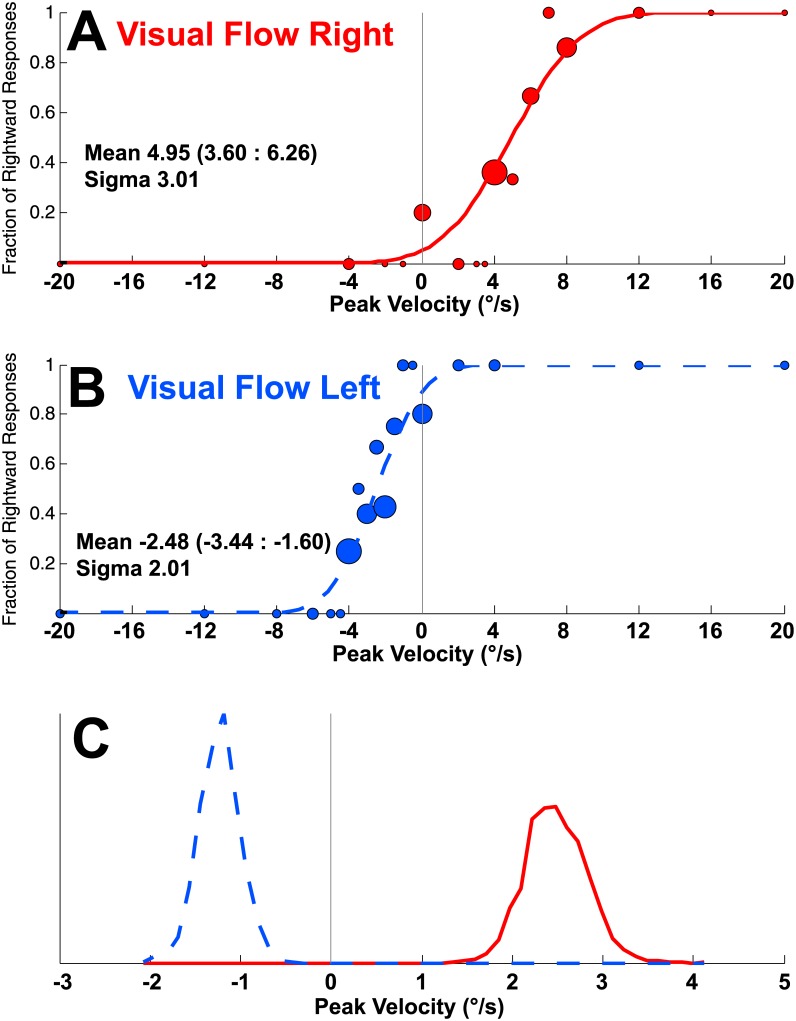
Sample data from an individual subject (#1) during a block of trials in which there was rotatory visual motion and a rotatory inertial stimulus. The circles in panels A&B represents the test stimuli given at a peak velocity with the diameter of the dot proportional to the number of responses at that stimulus level (the smallest dots represent a single stimulus and the largest 10 stimuli). The best fit cumulative distribution function (CDF) to the data is shown for each visual stimulus direction. (A) Responses to stimuli in which the visual stimulus moved to the right (implying leftward rotation in a fixed environment). (B) Responses to stimuli in which the visual stimulus moved left. The stimuli in panels A&B were randomly interleaved during the block of trials. (C) The CDF was fit to the data in A & B after being randomly resampled 2,000x. The mean was calculated for each iteration. The histograms of these means demonstrate no overlap between the two distributions.

Subject’s responses were fitted to a cumulative Gaussian function and confidence intervals were determined using a Monte Carlo maximum-likelihood criteria, [42, 43] as used by others [39, 40], and our laboratory previously [30–33, 35, 38, 44]. The data from each subject was resampled and fitted 100 times to allow for multiple estimates of the mean. Standard deviation was then generated and 95% confidence intervals were determined based on the upper and lower bounds using 95% of the estimates. The width of the psychometric function (sigma) indicated a measure of the reproducibility of the responses. Curves fit to a sample subject’s data are shown in Fig 2.

## Results

**Fig 3:**
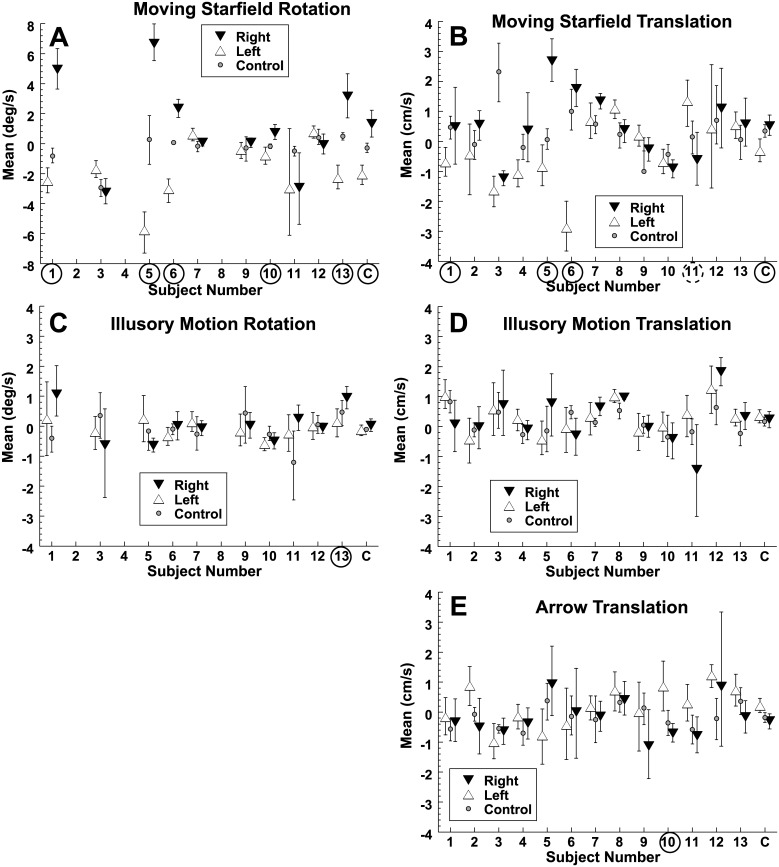
The effect of visual stimuli on inertial motion perception. In each case a small circle represents the control condition. Filled downward pointing triangles represent rightward visual motion (A,B), illusory motion (C,D), or a rightward pointing arrow (E). Open upward pointing triangles represent analogous leftward motion. Error bars represent the 95% confidence interval. The scale was expanded in panel A due to the larger effects in this condition. Subjects numbers are circled if there was a significant effect, dashed circles represent a significant effect in the direction opposite of expected. The furthest right column, (C) represents the combined or averaged response across subjects.

All subjects reported positively that they perceived an illusion of motion in the intended direction from the illusory motion visual stimuli (Fig 1). The point of subjective equality (PSE) for inertial movement in each subject and condition are shown (Fig 3).

Visual motion biased inertial motion perception in both yaw (Fig 3A) and sway (Fig 3B). The average bias was such that no inertial motion was more likely to be perceived in the direction opposite visual flow. This is what would be expected when the visual flow represents motion of a fixed environment relative to a moving observer. The mean difference between the two directions was -0.8 cm/s (sway) and -3.4°/s (yaw), with the negative value indicating that the bias was opposite the direction of visual motion (Table 1).

**Table 1:** Difference between the means from the left and right staircases from each block as measured in terms of peak velocity.

	Left-Right Difference	*P* value
Sway, Illusory motion	0.108 cm/s	0.555
Sway, Visual motion	-0.832 cm/s	0.001
Sway, Arrow	0.508 cm/s	0.02
Yaw, Illusory motion	-0.154°/s	0.285
Yaw, Visual motion	-3.394°/s	0.001

In contrast, illusory visual motion did not bias inertial motion perception with either rotation (Fig 3C) or translation (Fig 3D). Only one subject (#13 for rotation as shown in Fig 3C) had a convincing effect (*P* < 0.01, Monte Carlo method).

When an arrow was used to suggest a direction of motion, a small effect was observed. The average bias was 0.5 cm/s, which demonstrated a significant effect of arrow direction (*P* = 0.02, Monte Carlo method) when the subjects were combined. When the individual subjects were examined (Fig 3E) only one demonstrated a convincing effect (#10) and two (#5 and 6) had a trend towards a bias in the opposite direction.

Although the experimental protocol was optimized to measure the mean of the psychometric function (i.e., the PSE), the curve-fitting technique also estimated the threshold (sigma). For translation, the average threshold was 1.1 ± 0.6 (mean ± SD) cm/s and did not vary significantly (*P* > 0.05) based on the type of visual stimulus used. For rotation, the mean threshold was 1.1 ± 0.7°/s, which also did not vary based on the type of visual stimulus.

## Discussion

The results of this study indicate that our human subjects do not appear to have a significant bias in left right self-motion discrimination when exposed to illusory motion in the horizontal plane, as determined by sway translation or yaw rotation. However, when exposed to a moving visual field, our subjects did have a small but statistically significant shift in self-motion perception for both sway translation and yaw rotation. Although rotation and translation have different units, the effect appears to be greatest relative to the threshold of motion perception during yaw rotation. These biases tended to be small, and yaw rotation with a moving visual stimulus was the only condition in which inertial perception was biased by an amount greater than the perceptual threshold.

A fundamental challenge in selecting an appropriate illusory motion image was finding an image with discrete directionality. Most of the more well-known illusory motion images present motion in a two-dimensional plane, often times without a uniform direction of travel. Many two-dimensional RAPs based on the peripheral drift illusion [9] rotate either clockwise or counter clockwise, and neither of these directions are in phase with the desired directions of movement for our experiments. Studies have demonstrated that beyond RAP placement and color selection, contrast in direction itself within the image also enhances the overall illusion [45]. Because of this, single direction RAPs were not compelling enough for our purposes, although they have been used previously for fore-aft vection perception [16]. Our image was designed with the goal of having compelling illusory motion while establishing a discrete directionality. Although the direction of travel is consistent based on the orientation, it does consist of a complex stimulus with both rotation and translation around a torus. This complexity appeared to be a necessary shortcoming, as we were unable to design a similarly compelling illusory motion image that did not contain complex directionality. Because of the bi-axial nature of the stimulus, we tested both rotation and translation in an attempt to single out each movement in the horizontal plane. The consistency between both illusory motion conditions (i.e. the lack of a significant shift in the perceived direction of travel) helps to confirm that the illusory motion overall did not influence discrimination of self-motion direction.

It was thought that the illusory motion would provide at least some cognitive suggestion, which might bias responses. As a control for this we used an arrow in some trial blocks. The visible arrow had an effect on direction perception, but it was small and less than half the perceptual threshold. Because this was a forced-choice paradigm, subjects were required to report a direction if they were uncertain. Thus an arrow only influenced direction in the sub-threshold movements where subjects were essentially guessing. It has previously been shown that such cognitive bias can influence forced-choice experiments [46]. The current data demonstrate that susceptibility to this was variable between subjects, with some subjects having a tendency to report an opposite influence. But overall, the arrow had a significant and larger effect than the illusory motion stimuli, suggesting that the illusory motion may not even provide cognitive suggestion.

Another issue we encountered in dealing with illusory motion was quantifying the perceived velocity of the illusion. The velocity of travel for the illusory motion images has been shown to vary between observers, but similar to previous studies, [10] all of our subjects saw compelling illusory motion in the expected direction[10]. Using the work of Backus, et. al. we sought to create a compelling illusion by maximizing contrast with color and adjusting the proximity of the contrasted elements. After various iterations, we ultimately decided on the image seen in Fig 1. Increasing the number and density of the RAPs within the image and altering their layout helped to create more compelling motion. The final images were based on the input of the authors, artist, and feasibility trials with preliminary subjects who were not included in this study in order to avoid bias from previous exposure Because of the possibility of perceived differences in velocity and the inability to discretely match the velocity of the illusory motion images and the moving visual images, it is possible that the motion was not fast enough or consistent enough to influence self-motion perception in our subjects.

Another potential confounding issue was the duration of the stimulus used. The stimulus used was 8s, which was long enough to see a compelling vection effect with a visual motion stimulus. Some other studies have noted a mean onset of vection around 6s, [22, 47] although even in these studies some subjects had latencies that were 10s or longer. Another recent study, which used an inertial nulling task, found a more compelling vection effect at 15s relative to 7.5s and shorter [29]. However the stimulus used in that study was significantly different than in the current one in that it included optic flow and measured effects after the visual stimulus completed. A prior study on vection with illusory motion found that the latency could be less than 5s or longer than 20s depending on the flickering rate [16]. Thus it is possible that even though the current parameters did not produce a vection perception strong enough to bias inertial motion perception, other illusory visual stimuli or stimulus presentation parameters might show a bias. Given the forced-choice method used in the current experiments that required the stimulus to be presented multiple times within a trial block, extremely long stimulus presentations would be impractical because the trial block would become extended and it would be difficult to maintain subject alertness.

It is not clear how generalizable the current results are to illusory motion in general. For instance an ‘active volcano’ has recently been proposed as a method of inducing vection in the fore-aft direction[16]. Neither this stimulus nor this direction of self-motion was examined in the current study. There are also a large number of parameters that can potentially influence vection perception, including flickering, contrast, luminance, color, jitter, size, presence of audio stimuli, and the duration of stimulus presentation[1, 10, 18, 22, 47–49]. Although we feel we provided a compelling visual stimulus that gave an illusion of vection, it is possible that another combination of stimulus parameters might cause a larger bias in self-motion perception than reported in this study.

Overall, our data confirm that a moving visual field can influence self-motion perception in the horizontal plane for both sway translation and yaw rotation. Cognitive suggestion using an arrow had a sub-threshold but still significant effect. We did not find any significant shift in inertial motion perception when using an illusory motion image that simulated movement in the horizontal plane with both translational and rotational components. We also did not find any significant influence from higher cognitive suggestion alone, which helps to validate the results found with both the moving and illusory motion images.
